# Insights into the role of the junctional region of *Plasmodium falciparum* dihydrofolate reductase-thymidylate synthase

**DOI:** 10.1186/1475-2875-12-91

**Published:** 2013-03-12

**Authors:** Natpasit Chaianantakul, Rachada Sirawaraporn, Worachart Sirawaraporn

**Affiliations:** 1 Department of Biochemistry, Faculty of Science, Mahidol University, Bangkok, 10400, Thailand

**Keywords:** Malaria, *Plasmodium falciparum*, DHFR-TS, Junctional region

## Abstract

**Background:**

*Plasmodium falciparum* dihydrofolate reductase-thymidylate synthase (*pf*DHFR-TS) is a well-defined target of anti-malarial drug, such as pyrimethamine and cycloguanil. Emergence of malaria parasites resistant to these drugs has been shown to be associated with point mutations of the gene coding for the target enzymes. Although the 3D-structure of *P. falciparum* bifunctional *pf*DHFR-TS has been reported previously, relatively little is known about the interactions between the *pf*DHFR and *pf*TS domains and the roles of the junctional region that links the two domains together. Therefore, a thorough understanding of the interaction of the two domains and the role of the junctional region of this target is important as the knowledge could assist the development of new effective anti-malarial drugs aimed at overcoming drug-resistant malaria.

**Methods:**

A system was developed to investigate the interaction between *pf*DHFR and *pf*TS domains and the role of the junctional region on the activity of the recombinant *pf*TS. Based on the ability of co-transformed plasmids coding for *pf*DHFR and *pf*TS with truncated junctional region to complement the growth of TS-deficient *Escherichia coli* strain χ2913recA(DE3) on minimum media without thymidine supplementation, active *pf*TS mutants with minimal length of junctional region were identified. Interactions between active *pf*DHFR and the *pf*TS domains were demonstrated by using a bacterial two-hybrid system.

**Results:**

Using TS-deficient *E. coli* strain χ2913recA(DE3), the authors have shown for the first time that in *P. falciparum* a junctional region of at least 44 amino acids or longer was necessary for the *pf*TS domain to be active for the synthesis of thymidylate for the cells. Truncation of the junctional region of the bifunctional *pf*DHFR-TS further confirmed the above results, and suggested that a critical length of the junctional peptide of *pf*DHFR-TS would be essential for the activity of TS to catalyze the synthesis of thymidylate.

**Conclusion:**

The present study demonstrated the interactions between the *pf*DHFR and *pf*TS domains of the bifunctional *pf*DHFR-TS, and revealed that the junctional region linking the two protein domains is essential for the expression of catalytically active *pf*TS domain. The findings could be useful since inhibition of the *pf*DHFR-TS domain-domain interaction could form a basis for the development of new anti-malarial drugs based on targeting the non-active site region of this important enzyme.

## Background

Malaria remains an important disease in many tropical and sub-tropical countries [1]. The disease has become a global health threat, with over one million deaths annually, mostly children in sub-Saharan Africa [2]. The emergence of malaria resistance to almost all the currently available anti-malarial drugs has highlighted an urgent need to identify new malarial targets, and develop new effective drugs to combat the drug-resistant parasites [3-8]. *Plasmodium falciparum* dihydrofolate reductase-thymidylate synthase (*pf*DHFR-TS) is a well-defined target of antifolate drugs such as pyrimethamine and cycloquanil. The enzyme is responsible for the production of folates as well as thymidylate (dTMP) required for DNA synthesis [9]. Unfortunately, the emergence of anti-folate resistance has compromised the utility of the drugs and presented an urgent need to discover new drug targets and to develop novel effective drugs to combat drug-resistant parasites.

Structural studies of *P. falciparum* DHFR-TS revealed that the native enzyme is a homodimeric protein comprising 231 residues of DHFR domain (~27 kDa) at the N-terminus, followed by a short junctional region of 89 residues (~11 kDa) and 288 residues (~34 kDa) of the TS domain at the C-terminus of the protein [10,11]. It has been postulated that such a bifunctional arrangement could have evolved as a mechanism for the tight coupling generation of reduced folates required for the synthesis of amino acids, purine, pyrimidine, and dTMP, a phenomenon called “substrate channelling”. Support for this hypothesis comes from the evidence of metabolic channelling of the H_2_folate produced in the TS-catalyzed reaction, which was found to proceed at a faster rate than the diffusion rate [12]. Data from the bifunctional DHFR-TS of *Leishmania major* and *Toxoplasma gondii* also supported the substrate channelling hypothesis [12,13]. Nevertheless, the mechanism of substrate channelling for *pf*DHFR-TS remains unclear since, based on the structure of *pf*DHFR-TS, such a mechanism could not explain the delivery of H_2_folate from the *pf*TS domain to the active site of *pf*DHFR, and an electrostatic channelling mechanism was proposed as a possible alternative method [14-16].

The junctional region (JR) linked between the DHFR and TS domains in parasitic protozoa reported thus far varies significantly in length depending upon the source [9,10,16,17]. The long JR found in *P. falciparum* (89 amino acids) provides a number of interactions which facilitate contacts with the DHFR domain of the opposite half of the DHFR-TS dimer, and brings the two DHFR domains closer together. Structural alignments of DHFR-TS enzymes from *Cryptosporidium hominis* and *P. falciparum* revealed that the JR played an important role in the orientation of the DHFR domain relative to TS [17]. Therefore, inhibition of the interaction between the JR, the DHFR and TS domains could be a possible approach for the development of novel effective anti-malarial drugs [18]. The present study therefore describes an approach towards understanding the interactions between DHFR and TS domains of the bifunctional *pf*DHFR-TS, and the important role of JR on the activity of the TS domain. This is the first successful expression of the catalytically active *pf*TS domain that has JR attached at its N-terminus, as all attempts in the past to express *pf*TS domain failed. Through deletion of the JR, it is demonstrated that a critical length of JR is required for proper folding of the *pf*TS domain leading to an active molecule of *pf*TS. The findings highlight the importance of the JR which links DHFR and TS domains and suggest a possible alternative in exploiting the non-active site region of this important enzyme in developing new anti-malarial drugs to overcome drug-resistant parasites.

## Methods

### Materials

Restriction endonucleases and T_4_ DNA ligase were obtained from New England Biolabs, Life Technology, Inc. 2^′^-deoxyuridylate, 5-fluoro-2^′^- deoxyuridylate, and NADPH were from Sigma. MTX-sepharose CL-6B (~ 1 μmole/ml) [12], H_2_folate [19], and CH_2_H_4_folate [20] were prepared as described. Custom primer syntheses and DNA sequencing were from BioDesign Co Ltd and Genome Institute. TS-deficient *Escherichia coli* strain χ2913recA(DE3) was used for the genetic complementation studies to monitor the function of TS.

### Construction and transformation of recombinant plasmids

The gene coding for *P. falciparum* DHFR (amino acids 1–228) was amplified using a template DNA from pET*pf*DHFR-TS [21]. The *pf*JRTS mutants were constructed by the PCR mutagenesis method. The PCR reaction (100 μl) is composed of 50 ng pET*pf*DHFR-TS as a DNA template, 25 ρmole of each primer (Table 1), 200 μM of dNTPs, 1.5 mM MgCl_2_, and 2.5 units of *Taq* DNA polymerase in 1 x reaction buffer. The PCR conditions were as follows: 1 cycle of 94°C for 3 min, then 25 cycles of 94°C for 45 sec, annealing at 45°C for 30 sec, and extension at 72°C for 1 min. This was followed by a final extension at 72°C for 5 min. Deletion mutants of bifunctional *pf*DHFR-TS were constructed using the whole plasmid amplification PCR approach [22] employing recombinant plasmid pET15b carrying the *P. falciparum* DHFR-TS(3D7) gene as a template and sets of primers as shown in Table 2. The PCR products were analysed by agarose electrophoresis, and were further purified using a Qiaquick Gel Extraction kit.

**Table 1:** **Primers used for the construction of truncated *pf*JRTS mutants**

**Name**	**Length (bases)**	**Sequence (5**′ **⟶ 3**′**)**	**Utilities and description**
JRTSΔ232-235	28	AAAGAATCCC**ATG**GAACAAAATTGTATA	Sense strand PCR primer for the construction of pET-*pf*JRTSΔ232-235. Bold-face letters represent Met introduced in front of Glu^236^. Underlined sequence is the restriction site for *Nco*I.
JRTSΔ232-251	28	AAAGAATCCC**ATGGAA**AAGAATGATGAC	Sense strand PCR primer for the construction of pET-*pf*JRTSΔ232-251. Bold-face letters represent Met-Glu introduced infront of Lys^252^. Underlined sequence is the restriction site for *Nco*I.
JRTSΔ232-265	28	AAAGAATCCC**ATG**GAATTTTACAAAAAT	Sense strand PCR primer for the construction of pET-*pf*JRTSΔ232-265. Bold-face letters represent Met introduced in front of Glu^266^. Underlined sequence is the restriction site for *Nco*I.
JRTSΔ232-271	28	AAAGAATCCC**ATG**GACAAATATAAAATT	Sense strand PCR primer for the construction of pET-*pf*JRTSΔ232-271. Bold-face letters represent Met introduced in front of Asp^272^. Underlined sequence is the restriction site for *Nco*I
JRTSΔ232-274	57	CCCCTCTAGAAATAATTTTGTTTAACTTTAAGAAGGAGATATACC**ATG**AAAATTAAT	Sense strand PCR primer for theconstruction of pET-*pf*JRTSΔ232-274. Bold-face letters represent Met introduced in front of Lys^275^. Underlined sequence is the restriction site for *Xba*I
JRTSΔ232-276	57	CCCCTCTAGAAATAATTTTGTTTAACTTTAAGAAGGAGATATACC**ATG**AATTATGAA	Sense strand PCR primer for the construction of pET-*pf*JRTSΔ232-276. Bold-face letters represent Met introduced in front of Asn^277^. Underlined sequence is the restriction site for *Xba*I
JRTSΔ232-277	57	CCCCTCTAGAAATAATTTTGTTTAACTTTAAGAAGGAGATATACC**ATG**TATGAAAAT	Sense strand PCR primer to prepare construct pET-*pf*JRTSΔ232-277. Bold-face letters represent Met introduced in front of Tyr^278^. Underlined sequence is the restriction site for *Xba*I
JRTSΔ232-299	28	AAAGAATCCC**ATG**GAAGAGAAAAATAAA	Sense strand PCR primer to prepare construct pET-*pf*JRTSΔ232-299. Bold-face letters represent Met introduced in front of Glu^300^. Underlined sequence is the restriction site for *Nco*I
N3TS	22	ACTCATGGATCCTTAAGCAGCC	Antisense strand PCR primer for the construction of all pET-*pf*JRTS mutants. Underlined sequence is the restriction site for *BamH*I

**Table 2:** **Primers used for construction of truncated bifunctional *pf*DHFR-TS**

**Name**	**Length (bases)**	**Sequence (5**′ **⟶ 3**′**)**	**Utilities and description**
Δ229-277 Sense	60	ACAACATTGGATTTTATCATTTATAAG*AAA***TAT**GAAAATGATGATGATGATGAAGAAGAA	Sense strand PCR primer for the construction of *pf*DHFR-TSΔ229-277. Lys^228^ (italic) was followed by Tyr^278^(bold)
Δ229-277 Antisense	60	TTCTTCTTCATCATCATCATCATTTTCATATTTCTTATAAATGATAAAATCCAATGTTGT	Antisense strand PCR primer for the construction of *pf*DHFR-TSΔ229-277
Δ229-276 Sense	60	ACAACATTGGATTTTATCATTTATAAG*AAA***AAT**TATGAAAATGATGATGATGATGAAGAA	Sense strand PCR primer for the construction of *pf*DHFR-TSΔ229-276. Lys^228^ (italic) was followed by Asn^277^(bold)
Δ229-276 Antisense	60	TTCTTCATCATCATCATCATTTTCATAATTTTTCTTATAAATGATAAAATCCAATGTTGT	Antisense strand PCR primer to amplify *pf*DHFR-TSΔ229-276
Δ229-275 Sense	60	ACAACATTGGATTTTATCATTTATAAG*AAA***ATT**AATTATGAAAATGATGATGATGATGAA	Sense strand PCR primer for the construction of *pf*DHFR-TSΔ229-275. Lys^228^ (italic) was followed by Ile^276^(bold)
Δ229-275 Antisense	60	TTCATCATCATCATCATTTTCATAATTAATTTTCTTATAAATGATAAAATCCAATGTTGT	Antisense strand PCR primer to amplify *pf*DHFR-TSΔ229-275
Δ229-274 Sense	60	ACAACATTGGATTTTATCATTTATAAG*AAA***AAA**ATTAATTATGAAAATGATGATGATGAT	Sense strand PCR primer for the construction of *pf*DHFR-TSΔ229-274. Lys^228^ (italic) was followed by Lys^275^(bold)
Δ229-274 Antisense	60	ATCATCATCATCATTTTCATAATTAATTTTTTTCTTATAAATGATAAAATCCAATGTTGT	Antisense strand PCR primer to amplify *pf*DHFR-TSΔ229-274
Δ229-271 Sense	60	ACAACATTGGATTTTATCATTTATAAG*AAA***GAC**AAATATAAAATTAATTATGAAAATGAT	Sense strand PCR primer for the construction of *pf*DHFR-TSΔ229-271. Lys^228^ (italic) was followed by Asp^272^(bold)
Δ229-271 Antisense	60	ATCATTTTCATAATTAATTTTATATTTGTCTTTCTTATAAATGATAAAATCCAATGTTGT	Antisense strand PCR primer to amplify *pf*DHFR-TSΔ229-271
Δ229-265 Sense	60	ACAACATTGGATTTTATCATTTATAAG*AAA***GAA**TTTTACAAAAATGTAGACAAATATAAA	Sense strand PCR primer for the construction of *pf*DHFR-TSΔ229-265. Lys^228^ (italic) was followed by Glu^266^(bold)
Δ229-265 Antisense	60	TTTATATTTGTCTACATTTTTGTAAAATTCTTTCTTATAAATGATAAAATCCAATGTTGT	Antisense strand PCR primer to amplify *pf*DHFR-TSΔ229-265

The amplified product was cloned into pAC28 expression plasmid [23] to express catalytically active pAC*pf*DHFR. Likewise, sequences coding for truncated JR with the *pf*TS domain attached were amplified using the primers as listed in Table 1, and cloned into pET-15b to yield plasmids pET-*pf*JRTS△232-235, pET-*pf*JRTS△232-251, pET-*pf*JRTS△232-265, pET-*pf*JRTS△232-271, pET-*pf*JRTS△232-274, pET-*pf*JRTS△232-276, pET-*pf*JRTS△232-277, and pET-*pf*JRTS△232-299. These mutant pET-*pf*JRTS plasmids were transformed into electro-competent *E. coli* strain χ2913 cells by electroporation using pulses set at 1.8 kV, 400Ω, 25 μF and a pulse length of ~8-10 min. After centrifugation at 6,500 *g* at 4°C for 10 min and resuspending the cell pellets in 2 ml of 10% glycerol, a second electroporation was performed to transform 100 μl of the first transformed cells with ~50 ng of pAC-*pf*DHFR. After recovering the cells by addition of 0.9 ml of LB broth followed by vigorous shaking for 1 hour at 37°C, the cells were plated onto LB agar containing 100 μg/ml ampicillin and 30 μg/ml kanamycin. Colonies appeared on the plates after overnight incubation at 37°C were individually picked and grown overnight in 1 ml LB broth containing 100 μg/ml ampicillin and 30 μg/ml kanamycin at 37°C.

Truncation of JR of the bifunctional *pf*DHFR-TS was performed by PCR amplification using the whole recombinant plasmid pET15b carrying gene coding for the bifunctional *P. falciparum* DHFR-TS (3D7) as a template, and the primers as listed in Table 2. The resulting truncated mutants, i.e., *pf*DHFR-TSΔ229-265, *pf*DHFR-TSΔ229-271, *pf*DHFR-TSΔ229-274, *pf*DHFR-TSΔ229-275, *pf*DHFR-TSΔ229-276, and *pf*DHFR-TSΔ229-277, were used to co-transform pAC-28 plasmid in the complementation study.

### Genetic complementation studies

Overnight culture of the co-transformants was streaked on a minimal agar plate [24] containing 100 μg/ml ampicillin, 30 μg/ml kanamycin, and 0.025 mM IPTG without thymidine supplementation. The plates were incubated at 37°C for 48 hours. Colonies appearing on this plate were considered to have positive genetic complementation, and were selected for subsequent characterization and verification of the expressed enzymes.

### Expression and purification of the expressed enzymes

An overnight culture of *E. coli* χ2913 harbouring two plasmids, i.e., pAC-*pf*DHFR and truncated pET-*pf*JRTS with different lengths of truncated JR, was inoculated at 1% inoculum in LB containing 100 μg/ml of ampicillin, 30 μg/ml kanamycin. The bacteria were grown at 37°C until the OD_600_ of the cell suspension reached ~0.5-0.6, and isopropyl β-D thiogalactopyranoside (IPTG) was added at a final concentration of 0.025 mM. The culture was allowed to grow with shaking at 20°C for 20 hours. The cells were harvested by centrifugation at 6,500 *g* for 10 min at 4°C, washed once with 250 ml cold phosphate buffered saline, pH 7.4, resuspended in buffer A (20 mM potassium phosphate buffer, pH 7.0, 0.1 mM EDTA, 10 mM DTT, 20% glycerol) containing 0.2 M KCl, and passed through a French Pressure Cell (American Instruments Co Inc, USA) at 12,000 psi three times. After centrifugation at 20,000 *g* for 1 hour at 4°C, the clear supernatant of the crude sample was circulated at a flow rate of ~0.5 ml/min in a methotrexate-sepharose CL-6B column (1.5 × 5.0 cm) pre-equilibrated with buffer A containing 0.2 M KCl. After overnight circulation, the column was washed with 30 ml of buffer A containing 0.75 M KCl, followed by 20 ml of buffer A containing 0.2 M KCl. The column was then washed with 30 ml of elution buffer (50 mM TES pH 7.8, 0.1 mM EDTA, 10 mM DTT, 20% glycerol, 50 mM KCl) containing 4 mM H_2_folate to elute DHFR. Fractions of 1 ml were collected. Active fractions with DHFR activity were pooled, concentrated, and H_2_folate in the pooled fraction was removed by passing the pooled fraction through a pre-packed NAP-25 column (Pharmacia) pre-equilibrated with buffer A.

### Enzyme assays and protein analysis

The activity of DHFR was determined spectrophotometrically by monitoring the rate of decrease in absorbance at 340 nm [12,25]. The standard DHFR assay (1 ml) in a 1-cm path-length cuvette was composed of 100 μM H_2_folate, 100 μM NADPH, 50 mM TES, pH 7.0, 75 mM β-mercaptoethanol, 1 mg/ml bovine serum albumin, and ~0.01 units of enzyme. The reaction was initiated with H_2_folate. One unit of DHFR activity is defined as the amount of enzyme that produces 1 μmole of product per minute at 25°C.

The activity of TS was determined by monitoring the increase of absorbance at 340 nm due to the formation of H_2_folate at 25°C [26]. The reaction (1 ml) in 1-cm path-length cuvette was composed of 50 mM TES, pH 7.4, 25 mM MgCl_2_, 1 mM EDTA, 6.5 mM HCHO, 75 mM β-mercaptoethanol, 100 μM (6R) CH_2_H_4_folate, 125 μM dUMP and the enzyme. The reaction components, except for dUMP, were incubated at 25°C for at least 5 min to obtain the baseline prior to initiation the reaction with dUMP. One unit of TS activity is defined as the amount of enzyme that produces 1 nmole of product per minute at 25°C. The activities of the expressed enzymes were reported as mean ± standard deviation.

### [^3^H]-FdUMP binding assay

The ability of the *pf*JRTS mutants with truncated JR to form covalent complex with [^3^H]-FdUMP and CH_2_H_4_folate was investigated by incubating the mutant proteins with 0.5 μM [^3^H]-FdUMP (19.3 Ci/mmol), 0.1 mM CH_2_H_4_folate, and 6.5 mM formaldehyde in 50 mM TES pH 7.4, 25 mM MgCl_2_, 1 mM EDTA, and 75 mM β-mercaptoethanol for 15 min at room temperature. The reaction was then electrophoresed on 12.5% SDS-PAGE. After Coomassie-Blue staining and destaining, the gel was soaked with autoradiography enhancer (EN^3^HANCE™, New England Nuclear) with gentle shaking for 30 min at room temperature. This was followed by washing the gel with cold water and drying the gel on a piece of filter paper under vacuum at 80°C for 30 min. The dried gel was then exposed on AGFA X-ray film at -80°C with intensifying screens for three to five days before development of the X-ray film.

### Bacterial two-hybrid system

Interactions between *pf*DHFR domain and *pf*TS domain including those *pf*TS mutants with truncated JR were investigated using an *E. coli* two-hybrid system (Stratagene Inc) [27,28]. The sequence coding for *pf*DHFR domain was cloned into pBT (bait) vector to yield pBT-*pf*DHFR, whereas the *pf*JRTS was cloned into pTRG (target) vector to yield the pTRG-*pf*JRTS plasmid. The two plasmids were co-transformed into BacterioMatch two-hybrid system reporter strain *E. coli* XL1-blue MRF’. Positive interaction is indicated by the ability of the bacteria to grow on LB agar plate supplemented with 250 μg/ml carbenicillin, 15 μg/ml tetracycline, 34 μg/ml chloramphenical, and 50 μg/ml kanamycin (LB-CTCK).

## Results

### Construction of *pf*JRTS and *pf*DHFR-TS mutants and growth complementation studies

Unlike the DHFR domain of the bifunctional *pf*DHFR-TS, which can be heterologously expressed to yield the catalytically active form of enzyme [29-32], all attempts to express the catalytically active domain of *pf*TS have failed. Evidence from deletion of *pf*DHFR-TS suggested that the amino terminus of the *pf*DHFR domain is important for the function of the *pf*TS domain, and interactions between the *pf*DHFR and *pf*TS domains are important [18]. To address the important function of JR, mutant constructs of the *pf*JRTS domain with various lengths of JR sequences were constructed and co-transformed with plasmid expressing the catalytically active *pf*DHFR domain (pAC-*pf*DHFR) in TS-deficient *E. coli* strain χ2913recA(DE3). Successful expression of catalytically active *pf*TS was monitored by the ability of the co-transformed bacteria to complement growth upon plating on minimum agar plate and the detectable TS activity in the crude extract of the cells.

**Figure 1:**
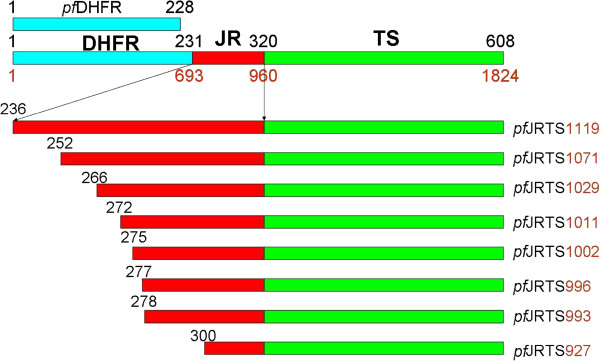
**Schematic diagram representing full-length *pf*DHFR-TS, *pf*DHFR domain, and *pf*TS domains with truncated junction region (JR).** Gene encoding amino acid residues 1–228 of *pf*DHFR domain was cloned into pAC28 expression vector, whereas gene fragments encoding the *pf*TS domain with different lengths of JR that are attached to the N-terminus (amino acid residue 320) of *pf*TS were cloned into the pET15b expression vector. The numbers of amino acid are shown above the gene and the names of the mutants are indicated at the right.

**Figure 2:**
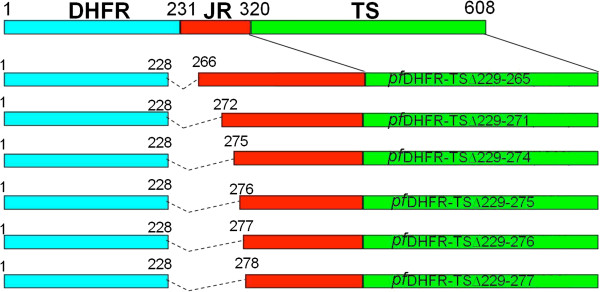
**Schematic diagram representing full-length and truncated *pf*DHFR-TS.** Constructs of *pf*DHFR domain containing amino acids 1–228 followed by truncated JR sequences of different lengths linked to the N-terminus of the *pf*TS domain were cloned into the pET15b expression vector. The numbers of amino acid are shown above the gene and the names of the mutants are indicated.

**Figure 3:**
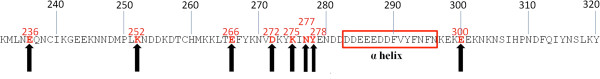
**Full-length sequence of *Plasmodium falciparum *JR and the sequences of truncated *pf*JRTS mutants.** The 89 amino acids of the full-length sequence of *P. falciparum* JR are shown, with amino acid numbers in relation to the full-length bifunctional *pf*DHFR-TS indicated above the sequence. Arrows indicate the amino acids corresponding to the first amino acid of the truncated ***pf*JRTS** constructs. The region where the α-helix is located is marked in the box.

**Figure 4:**
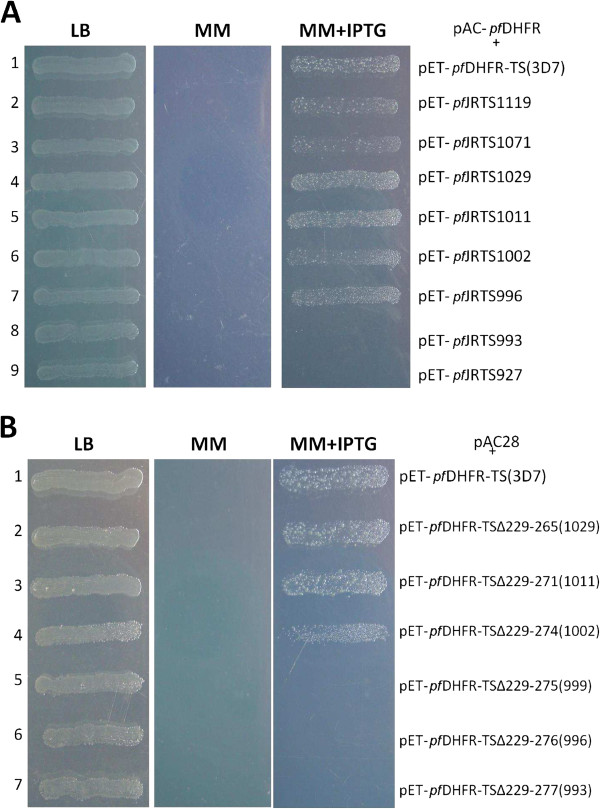
**Growth complementation of TS-deficient Escherichia coli cells harbouring.** (**A**) pAC-pfDHFR co-transformed with pET-pfJRTS containing truncated JR. (**B**) pAC28 co-transformed with pET-pfDHFR-TS with truncated JR. Cells from overnight culture were streaked on Luria-Bertani (LB), minimum media (MM), and minimum media supplemented with 0.025 mM IPTG (MM + IPTG). pET-pfDHFR-TS (3D7) was used as a controlled plasmid.

Figure 1 illustrates the constructs of *pf*JRTS mutants containing varying lengths of JR attached to the C-terminus of the *pf*DHFR domain (Asn^231^). The mutant with the longest JR (pET-*pf*JRTS△232-235) had only four amino acids of the JR (Lys^232^-Asn^235^) deleted, while mutants with the shortest JR (pET-*pf*JRTS△232-299) had 68 residues (Lys^232^-Lys^299^) removed. A similar approach was undertaken to construct bifunctional *pf*DHFR-TS deletion mutants of which the JR sequence was shortened to compare the effects of JR deletion with the truncated mutants of *pf*JRTS constructs. The bifunctional *pf*DHFR-TS deletion mutants being constructed include pET-*pf*DHFR-TS△229-265, pET-*pf*DHFR-TS△229-271, pET-*pf*DHFR-TS△229-274, pET-*pf*DHFR-TS△229-275, pET-*pf*DHFR-TS△229-276, and pET-*pf*DHFR-TS△229-277 (Figure 2). Figure 3 shows the 89 amino acids of *P. falciparum* JR sequence and indicates the positions of amino acids that were chosen to prepare truncated and deletion mutants. Results from growth complementation monitored after incubation of the plates overnight at 37°C revealed that pET-*pf*JRTS△232-235, pET-*pf*JRTS△232-251, pET-*pf*JRTS△232-265, pET-*pf*JRTS△232-271, pET-*pf*JRTS△232-274, and pET-*pf*JRTS△232-276 with corresponding JR length of 85, 69, 55, 49, 46, 44 amino acid residues, respectively, could grow on minimum media (Figure 4A, lanes 2–7), while pET-*pf*JRTS△232-277 and pET-*pf*JRTS△232-299 which had the JR length of 43 and 21 residues, respectively, did not show growth complementation (Figure 4A, lanes 8–9). The results suggest that the length of JR was important for the proper folding of the TS domain and hence affected its function. The data revealed that at least 44 residues (the mutant pET-*pf*JRTS△232-276) were required in the case of JR of *P. falciparum* in order for the *pf*TS domain to function properly.

As with the result for the construct *pf*JRTS TSΔ229-277, TS-deficient *E. coli* strain χ2913 recA(DE3) transformed with pET-*pf*DHFR-TS△229-277and pAC-28 could not grow on a minimum agar plate (Figure 4B, lane 7). Therefore, *pf*DHFR-TS mutants with a longer JR sequence, i.e. pET-*pf*DHFR-TS△229-265, pET-*pf*DHFR-TS△229-271, pET-*pf*DHFR-TS△229-274, pET-*pf*DHFR-TS△229-275, and pET-*pf*DHFR-TS△229-276, were constructed and tested for growth complementation. Complementation studies revealed that the constructs pET-*pf*DHFR-TS△229-265, pET-*pf*DHFR-TS△229-271, and pET-*pf*DHFR-TS△229-274 were able to grow on both LB agar and minimum agar supplemented with 0.025 mM IPTG (Figure 4B, lanes 2–4), whereas the construct pET-*pf*DHFR-TS△229-275 and pET-*pf*DHFR-TS△229-276 failed to show complementation (Figure 4B, lanes 5–6). The results using deletion mutants of bifunctional *pf*DHFR-TS are in good agreement with those from co-transformation of plasmids encoding *pf*JRTS with varying lengths of JR and plasmid expressing catalytically active *pf*DHFR.

### [^3^H]-FdUMP binding studies of *pf*JRTS and *pf*DHFR-TS mutant constructs expressing *pf*TS

**Figure 5:**
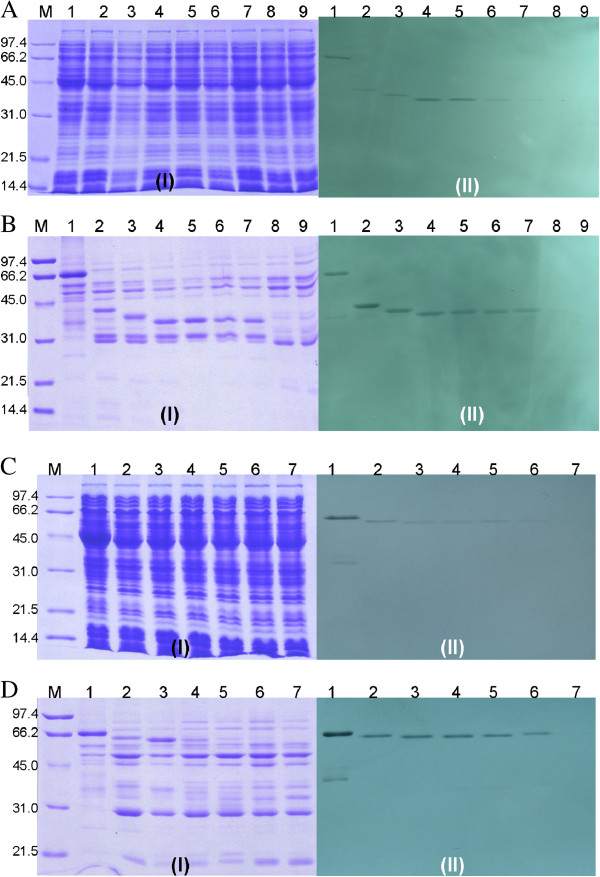
**[**^**3**^**H]-FdUMP binding of crude extracts and partially purified proteins.** Crude extracts and partially purified proteins from plasmids encoding pfDHFR co-transformed with pfJRTS mutants with truncated JR (panels **A** and **B**, respectively), and plasmids encoding pAC28 co-transformed with pfDHFR-TS with truncated JR (panel **C** and **D**, respectively) were labeled with [^3^H]-FdUMP. For panels **A** and **B**: lane 1, *pf*DHFR-TS (3D7); lane 2, *pf*JRTS△232-235 co-expressed with pAC28-*pf*DHFR; lane 3, *pf*JRTS△232-251 co-expressed with pAC28-*pf*DHFR; lane 4, *pf*JRTS△232-265 co-expressed with pAC28-*pf*DHFR; lane 5, *pf*JRTS△232-271 co-expressed with pAC28-*pf*DHFR; lane 6, *pf*JRTS△232-274co-expressed with pAC28-*pf*DHFR; lane 7, *pf*JRTS△232-276 co-expressed with pAC28-*pf*DHFR; lane 8, *pf*JRTS△232-277 co-expressed with pAC28-*pf*DHFR; lane 9, *pf*JRTS△232-299 co-expressed with pAC28-*pf*DHFR. For panels **C** and **D**: lane 1, *pf*DHFR-TS (3D7); lane 2, *pf*DHFR-TS△229-265; lane 3, *pf*DHFR-TS△229-271; lane 4, *pf*DHFR-TS△229-274; lane 5, *pf*DHFR-TS△229-275; lane 6, *pf*DHFR-TS△229-276; lane 7, *pf*DHFR-TS△229-277993. M is the molecular weight standard markers. (I) Coomassie blue stained SDS-PAGE, (II) autoradiogram of the SDS gel.

[^3^H]-FdUMP binding studies were carried out to monitor the expression of catalytically active *pf*TS from truncated *pf*JRTS mutants and the *pf*DHFR-TS deletion mutants found to show growth complementation on minimum media. Crude extracts from *E. coli* χ2913 co-transformed with plasmid expressing *pf*DHFR and mutant *pf*JRTSs with varying length of JR were subjected to SDS-PAGE (Figure 5A(I) and B(I)) and the gel was exposed to X-ray film (Figure 5A(II) and B(II)). In agreement with the results from genetic complementation results, co-transformation of pAC-*pf*DHFR and pET-*pf*JRTS△232-235, pET-*pf*JRTS△232-251, pET-*pf*JRTS△232-265, pET-*pf*JRTS△232-271, pET-*pf*JRTS△232-274 and pET-*pf*JRTS△232-276 formed [^3^H]-FdUMP-enzyme complexes which could be visualized on X-ray film as a band according to the size predicted from that of *pf*JRTS (Figure 5B, lanes 2–7). Co-transformation of pAC-*pf*DHFR and *pf*JRTS△232-277 and *pf*JRTS△232-299, however, showed relatively weak signal from [^3^H]-FdUMP-enzyme complexes (Figure 5B, lanes 8–9).

To investigate the role of JR in bifunctional *pf*DHFR-TS, formation of [^3^H]-FdUMP-enzyme complexes were monitored from the extracts of *E. coli* cells co-transformed with pAC28 plasmid and pET-*pf*DHFR-TS△229-265, pET-*pf*DHFR-TS△229-271, pET-*pf*DHFR-TS△229-274, pET-*pf*DHFR-TS△229-275, and pET-*pf*DHFR-TS△229-276 (Figure 5C and 5D). The results showed that both crude extracts and partially purified proteins of these constructs could form complexes with [^3^H]-FdUMP, though growth complementation could not be observed from pET-*pf*DHFR-TS△229-275, pET-*pf*DHFR-TS△229-276, and pET-*pf*DHFR-TS△229-277 (Figure 5D, lanes 5–7).

### Purification and characterization of the expressed enzymes

Crude extracts of the transformed TS-deficient *E. coli* that were both positive and negative from the growth complementation studies were assayed for DHFR and TS activities. Extracts that were positive were passed through a MTX-affinity column for purification of the *pf*DHFR domain [32]. The results from enzyme assays revealed that the specific activity of *pf*DHFR from all the co-transformants were not significantly different among the crude extracts (average 36.2 ± 3.1 nmole/min/mg), but the activity was only about half of that observed from the crude extract of the bifunctional *pf*DHFR-TS (Table 3). The specific activities of the partially purified *pf*DHFR upon affinity purification were 1,371 ± 208 nmole/min/mg (Table 3), about 3 times lower than that obtained from the purified *pf*DHFR-TS. Interestingly, the TS activities of the purified enzymes from the truncated *pf*JRTS mutants are in most cases 1–2 times higher than that from the purified *pf*DHFR-TS. The activity of *pf*TS was found to co-elute with that of *pf*DHFR, suggesting that the expressed *pf*TS domain was somehow associated with the *pf*DHFR domain. As indicated in Table 3, the TS activity of the *pf*JRTS mutants was found to decrease upon shortening the length of JR in the construct.

**Table 3:** **DHFR and TS activities in crude extracts and purified enzymes from TS-deficient *Escherichia coli *χ2913 harbouring pAC- *pf *DHFR and pET- *pf *JRTS with different lengths of JR**

**Specific activity**				
**Plasmid**	Crude extract*		Purified enzyme**	
	**(nmole/min/mg)**		**(nmole/min/mg)**	
	**DHFR**	**TS**	**DHFR**	**TS**
1. pET-*pf*DHFRTS (3D7)	70.3 ± 3.6	3.7 ± 0.4	5331.9 ± 113.8	99.0 ± 1.0
2. pAC-*pf*DHFR + pET-*pf*JRTSΔ232-235	39.5 ± 5.8	2.6 ± 0.2	1511.1 ± 227.5	164.1 ± 22.6
3. pAC-*pf*DHFR + pET-*pf*JRTSΔ232-251	34.2 ± 5.8	2.1 ± 0.4	1394.5 ± 275.7	134.4 ± 8.9
4. pAC-*pf*DHFR + pET-*pf*JRTSΔ232-265	37.8 ± 5.3	3.8 ± 0.5	1668.4 ± 234.7	132.0 ± 7.4
5. pAC-*pf*DHFR + pET-*pf*JRTSΔ232-271	39.9 ± 7.3	3.9 ± 0.7	1601.7 ± 206.1	107.9 ± 23.3
6. pAC-*pf*DHFR + pET-*pf*JRTSΔ232-274	38.3 ± 5.6	1.5 ± 0.3	1328.8 ± 231.7	70.1 ± 0.2
7. pAC-*pf*DHFR + pET-*pf*JRTSΔ232-276	34.2 ± 9.1	1.1 ± 0.1	1140.1 ± 137.8	44.2 ± 7.5
8. pAC-*pf*DHFR + pET-*pf*JRTSΔ232-277	31.0 ± 8.1	ND	1136.5 ± 132.0	ND
9. pAC-*pf*DHFR + pET*pf*JRTSΔ232-279	34.9 ± 7.2	ND	1193.6 ± 111.8	ND
10. pAC-28 + pET-15b	7.7 ± 0.4	ND	-	-

* Average values from three independent experiments.

**Average values from two independent experiments.

ND: not detectable.

The importance of the length of the JR sequence on the activity of *pf*TS was also investigated using the bifunctional *pf*DHFR-TS. Site-directed mutagenesis of the bifunctional *pf*DHFR-TS was performed to yield mutants containing the same lengths of JR as for the *pf*JRTS mutant constructs described above. These mutants include pET-*pf*DHFR-TS△229-265, pET-*pf*DHFR-TS△229-271, pET-*pf*DHFR-TS△229-274, pET-*pf*DHFR-TS△229-275, pET-*pf*DHFR-TS△229-276, and pET-*pf*DHFR-TS△229-277. Table 4 summarizes the *pf*DHFR and *pf*TS activities of the bifunctional DHFR-TS and the bifunctional mutants from the crude extracts and upon partial purification. In agreement with the growth complementation experiments, no *pf*TS activity was detected from the crude extracts and partial purification of pET-*pf*DHFR-TS△229-275, pET-*pf*DHFR-TS△229-276, and pET-*pf*DHFR-TS△229-277 mutants. However, the activities of the *pf*DHFR domain of these mutants remained active, though they were found to gradually decrease upon shortening the length of the JR sequence.

**Table 4:** **DHFR and TS activities in crude extracts and purified enzymes from TS-deficient *Escherichia coli *χ2913 harbouring pET- *pf *DHFR-TS and pET- *pf *DHFR-TS constructs with different lengths of JR**

**Specific activity**				
**Plasmid**	Crude extract*		Purified enzyme**	
	**(nmole/min/mg)**		**(nmole/min/mg)**	
	**DHFR**	**TS**	**DHFR**	**TS**
1. pET-*pf*DHFR-TS (3D7)	70.3 ± 3.6	3.7 ± 0.4	5331.9 ± 113.8	99.0 ± 1.0
2. pET-*pf*DHFR-TSΔ229-265	64.7 ± 5.8	1.1 ± 0.3	4583.2 ± 228.9	44.8 ± 1.9
3. pET-*pf*DHFR-TSΔ229-271	75.5 ± 12.4	1.0 ± 0.3	3844.6 ± 130.6	40.9 ± 5.9
4. pET-*pf*DHFR-TSΔ229-274	74.4 ± 18.5	0.9 ± 0.2	3487.8 ± 213.2	35.8 ± 2.4
5. pET-*pf*DHFR-TSΔ229-275	66.7 ± 7.3	ND	4719.3 ± 391.3	ND
6. pET-*pf*DHFR-TSΔ229-276	83.9 ± 1.0	ND	3357.8 ± 443.3	ND
7. pET-*pf*DHFR-TSΔ229-277	82.1 ± 3.0	ND	3137.8 ± 210.3	ND
8. pET-15b	3.1 ± 0.1	ND	-	-

* Average values from three independent experiments.

** Average values from two independent experiments.

ND: not detectable.

### Demonstration of *pf*DHFR-TS domain-domain interaction by *Escherichia coli* two-hybrid system

The interaction between the *pf*DHFR domain and *pf*JRTS was demonstrated using *E. coli* BacterioMatch™ two-hybrid system (Strategene). The *pf*DHFR domain was cloned into *Not*I-*BamH*I sites of a pBT bait plasmid, resulting in a pBT-*pf*DHFR plasmid, which contains the *pfdhfr* gene fused at the end of the bacteriophage *λcI* gene. The *pf*JRTS domain was cloned into *BamH*I-*Xho*I sites of the pTRG target plasmid, resulting in constructs that express truncated *pf*JRTS domains fused with the α-subunit of RNA polymerase. If the *pf*JRTS domain could interact with the *pf*DHFR domain, then this would stabilize the binding of RNA polymerase located close to the promoter and activate the transcription of the Amp^R^ reporter gene. As a consequence, the transformed reporter strain (*E. coli* XL1-blue MRF’) showed growth on Luria-Bertani agar containing carbenicillin, tetracycline, chloramphenical and kanamycin (LB-CTCK).

**Figure 6:**
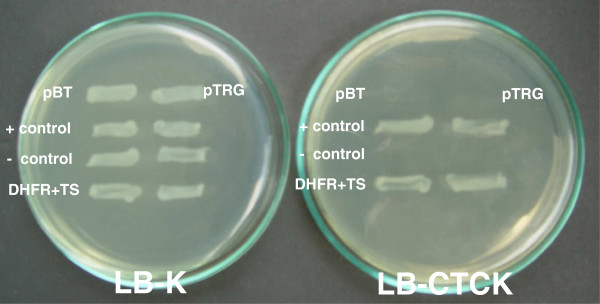
***Escherichia coli *two-hybrid showing interaction between *pf*DHFR and *pf*TS domains.***Escherichia coli* BacterioMatch™ two-hybrid system (Strategene) was employed for the study of the domain-domain interaction between *P. falciparum* DHFR and TS domains. The gene coding for *pf*DHFR was cloned into the bait plasmid pBT, whereas the gene for *pf*JRTS was cloned into the target plasmid pTRG. The two recombinant plasmids were transformed into the *E. coli* XL1-blue MRF’ reporter strain. Positive control is *E. coli* XL1-blue MRF’ co-transformed with pBT-LDF2 and pTRG-Gal11^p^ provided by the manufacturer. Negative control is *E. coli* XL1-blue MRF’. Agar plate on the left is LB-kanamycin (LB-K) and that on the right is LB- carbenicillin, tetracycline, chloramphenical and kanamycin (LB-CTCK).

The interaction between the DHFR monomer and the donated helix within the JR sequence has earlier been noted from the structures of the bifunctional DHFR-TS enzymes of *C. hominis* and *P. falciparum*[17,33]. An interaction of the *pf*DHFR and *pf*JRTS domains was demonstrated using the *E. coli* two-hybrid system [27,28,34]. The *E. coli* reporter strain co-transformed with plasmids pBT-*pf*DHFR and pTRG-*pf*JRTS (with full-length JR sequence) can grow on a LB-CTCK agar plate (Figure 6). The data is in agreement with the complementation results and results from [^3^H]-FdUMP binding studies supporting the importance of JR on the folding of the *pf*TS domain.

## Discussion

The JR represents a junctional region linked between the DHFR and the TS domain of parasitic protozoa. This region has been proposed as potential target for drug development in many parasitic protozoa. Indeed, this region in *C. hominis* from one monomer was reported to make extension contacts with the DHFR active site of the other monomer [17]. In *P. falciparum*, the amino acid residues Asp^283^-Asn^296^ of JR are strongly predicted to be involved in domain-domain interaction. Unfortunately, the major portions of JR (Lys^232^-Asn^280^) were not seen in the crystal structure previously reported [33]. Therefore, it remains unclear whether each DHFR domain of *P. falciparum* is linked to the TS domain as seen in *C. hominis* or there is a domain swapping assembly. The role of JR was characterized with respect to interdomain interaction.

A series of truncated mutants of *pf*JRTS were constructed containing varying lengths of JR and their interactions with the active *pf*DHFR domain were examined. By employing TS-deficient *E. coli* χ2913 and monitoring its growth complementation in minimum media without thymidine supplementation, the study showed that the *pf*JRTS construct with the shortest length of JR that could still show growth complementation in TS-deficient *E. coli* χ2913 was *pf*JRTS△232-276. The specific activity of DHFR determined for the crude extract of the monofunctional *pf*DHFR upon co-expression with *pf*JRTS△232-276 was 34.2 ± 9.1 nmole/min/mg, a value which was about half that obtained from the bifunctional *pf*DHFR-TS (70.3 ± 3.6 nmole/min/mg), but was about the same level for all truncated *pf*JRTS mutants. However, the specificity of TS of this truncated construct was only 1.1 ± 0.1 nmole/min/mg (Table 3), which is about 29% of the wild-type enzyme (3.7 ± 0.4 nmole/min/mg). It is noteworthy that the DHFR specific activities among the co-transformants investigated were comparable whereas the TS specific activities were dramatically reduced upon shortening of the JR sequence. The *pf*DHFR and *pf*JRTS domains could be co-purified by using a MTX-affinity column, suggesting that the *pf*DHFR and *pf*TS domains interacted with each other because methotrexate binds only to the *pf*DHFR domain. For the co-transformants that lose the *pf*TS activity including *pf*JRTS△232-277 and *pf*JRTS△232-299, the constructs still expressed active *pf*DHFR (Table 3). The expression of catalytically active *pf*TS upon cotransformation with *pf*DHFR plasmid was further confirmed by showing a positive autoradiogram of covalent complex formed as a result of [^3^H]-FdUMP bound to the expressed *pf*TS from the *pf*JRTS△232-276 mutant construct.

These results agree well with the previous studies, suggesting that the *pf*DHFR domain is essential for *pf*TS to be active [18,35]. However, one interesting piece of data from this laboratory showed that co-expression of the death mutant *pf*DHFR (Ser^108^ to Trp^108^ mutation) [36] and *pf*JRTS△232-235 or *P. vivax* DHFR with *pf*JRTS△232-235 could not confer expression of catalytically active *pf*TS (data not shown). This finding suggests that not only minimal length of JR is required but active *pf*DHFR domain is also important for the *pf*TS domain to be active. Although endogenous DHFR from an *E. coli* host exists, the enzyme could not promote the growth of TS-deficient *E. coli*. It is possible that amino acids within the *pf*DHFR domain are critically necessary to stabilize the DHFR tertiary structure which could indirectly influence the correct folding and hence the activity of the *pf*TS domain.

The domain-domain interaction of the bifunctional *pf*DHFR-TS was further demonstrated using an *E. coli* two hybrid system and the results obtained were consistent with other experiments. The pBT-*pf*DHFR and pTRG-*pf*JRTS co-transformant showed interaction and turned on the Amp^R^ gene, allowing the growth of *E. coli* XL1-blue MRF’. The results suggest that the minimal length of JR which could allow expression of the catalytically active *pf*TS was 44 amino acids, approximately half the full length of JR of the *P. falciparum* DHFR-TS.

The importance of the length of JR was investigated by the ability to complement the growth of TS-deficient *E. coli* in the absence of thymine of co-transformed plasmids harbouring the genes for *pf*DHFR and truncated *pf*JRTS compared to plasmid harbouring the gene for the bifunctional *pf*DHFR-TS. Complementation experiments support the conclusion that the length of JR sequence is crucial. The finding that positive complementation was still observed from the construct of truncated bifunctional *pf*DHFR-TS△229-274 which possesses two more amino acid residues than the case of monofunctional *pf*JRTS△232-276 could be due to the more complex structure of the bifunctional protein as compared to the monofunctional enzymes resulting from the interaction, or associated with the sensitivity of the complementation assay system. To further address this discrepancy, a more sensitive [^3^H]-FdUMP binding assay method was used to confirm that *pf*DHFR-TS△229-276 is the construct with the shortest length of JR that could still express active *pf*TS. The result was in good agreement with the results from complementation studies using monofunctional *pf*JRTS△229-276. The fact that the bifunctional mutant failed to show complemention in TS-deficient *E. coli* but could not detect the activity of *pf*TS by *in vitro* enzymatic assay could be due to the insufficient expression of protein in combination with the sensitivity of the complemention method, since a minimum TS activity of ~1-5 nmole/min/mg is necessary for complementation to be observed in *E. coli* χ2913 [37]. It should also be noted that the low expression observed in the truncated bifunctional *pf*DHFR-TS was consistent with a previous mutation study which proposed that the significant intracellular proteolysis activity associated with the α-helix structure in this region could be attributed to the low expression of the mutant proteins [38].

The present results clearly demonstrated that shortening the JR sequence in the *pf*DHFR-TS could affect the activity of both *pf*TS and *pf*DHFR. Contrary to that observed in *T. gondii* and *C. hominis* which have longer JR sequences, mutations of the JR sequence were reported to affect only the activity of DHFR but not the activity of TS [39,40]. This and other data support the conclusion that the length of JR sequence is necessary for the activity of both *pf*DHFR and *pf*TS domains, and hence highlights the importance of the JR sequence in *P. falciparum* bifunctional DHFR-TS.

It was not so surprising that the constructs *pf*JRTS△232-277 and *pf*DHFR-TS△229-277 showed no expression of catalytically active *pf*TS, despite the fact that the constructs contain a sequence that encompasses Asp^283^-Asn^296^, the region previously predicted to form α-helix and responsible for electrostatic interaction between the negatively charge residues and positive charge of the *pf*DHFR and *pf*TS from different domains [33]. A number of possible explanations could be as follows: (i) the length of JR in the constructs is too short, and this could affect the formation of a stable helix and consequently disturb the interaction with *pf*DHFR and interfere with the proper formation of *pf*TS conformation; (ii) too short a JR sequence could affect the structure which could result in a steric constraint or functional infringement of one domain upon the other; (iii) the existence of translational autoregulation could block translation of mRNA coding for bifunctional enzyme from binding to the DHFR domain of the protein [41]; and, (iv) the mutation could cause exposure of some parts of the enzyme which are sensitive to proteolysis [38]. Therefore, too short a JR sequence could neither support interaction between *pf*DHFR and *pf*TS domains nor contribute to proper folding of the *pf*DHFR-TS bifunctional protein.

## Conclusions

The data presented in this study show that: a) the presence of an active *pf*DHFR domain and the appropriate length of JR are critical for *pf*TS to fold correctly to be catalytically active; and, b) deletion of JR affects both the activities of the *pf*TS and the *pf*DHFR domain of the bifunctional *pf*DHFR-TS. From this study, the JR of *P. falciparum* has a unique role which is different from other long linker parasitic protozoa. The data described here could be useful for the development of compounds that bind to the JR and interfere with the dimerization of the *pf*TS subdomains. These studies could potentially lead to novel means of development of inhibitors targeting the JR sequence of the parasite.

## Abbreviations

DHFR-TS: Dihydrofolate reductase-thymidylate synthase; SHMT: Serine hydroxymethyltransferase; dTMP: 2^′^-deoxythymidine 5^′^-monophosphate; [3H]-FdUMP: Tritiated-5-fluoro-2^′^-deoxyuridine 5^′^-monophosphate; JR: Junctional region; MM: Minimal media; MTX: Methotrexate

## Competing interests

The authors declare that they have no competing interests.

## Authors’ contributions

NC participated in performing experiments and drafted the manuscript. RS helped to perform the experiments and discussion. WS conceived the study, participated in its design, data analysis and critical revision of the manuscript. All authors read and approved the final manuscript.
